# Parental obesity-induced changes in developmental programming

**DOI:** 10.3389/fcell.2022.918080

**Published:** 2022-10-07

**Authors:** Laura R. Cechinel, Rachael A. Batabyal, Robert J. Freishtat, Irene E. Zohn

**Affiliations:** Center for Genetic Medicine Research, Children’s National Hospital, Washington, DC, United States

**Keywords:** developmental programming, obesity, extracellular vesicles, placentation, adipogenesis, pancreatic developmnent, appetite regulation, epigenteics

## Abstract

Many studies support the link between parental obesity and the predisposition to develop adult-onset metabolic syndromes that include obesity, high blood pressure, dyslipidemia, insulin resistance, and diabetes in the offspring. As the prevalence of obesity increases in persons of childbearing age, so does metabolic syndrome in their descendants. Understanding how parental obesity alters metabolic programs in the progeny, predisposing them to adult-onset metabolic syndrome, is key to breaking this cycle. This review explores the basis for altered metabolism of offspring exposed to overnutrition by focusing on critical developmental processes influenced by parental obesity. We draw from human and animal model studies, highlighting the adaptations in metabolism that occur during normal pregnancy that become maladaptive with obesity. We describe essential phases of development impacted by parental obesity that contribute to long-term alterations in metabolism in the offspring. These encompass gamete formation, placentation, adipogenesis, pancreas development, and development of brain appetite control circuits. Parental obesity alters the developmental programming of these organs in part by inducing epigenetic changes with long-term consequences on metabolism. While exposure to parental obesity during any of these phases is sufficient to alter long-term metabolism, offspring often experience multiple exposures throughout their development. These insults accumulate to increase further the susceptibility of the offspring to the obesogenic environments of modern society.

## Introduction

The obesity epidemic is becoming one of the most significant public health challenges worldwide. The incidence of obesity has nearly tripled since 1975 (WHO, 2021). In 2016, 39% of adults over 18 years of age were overweight (1.9 billion adults), and 13% of adults were affected by obesity (650 million adults) (WHO, 2021). The number of children that are overweight or obese is rising, affecting 340 million children between the ages of 5–19 and 39 million children under the age of 5 worldwide (WHO, 2021). Likewise, obesity among women of childbearing age is increasing. For instance, in the United States, maternal obesity has increased by 14% since the 1990s (Poston, et al., 2016; Driscoll, 2020).

The idea that maternal nutritional status during early life can influence the long-term health of the offspring is now widely accepted (Godfrey et al., 2010; Hardikar et al., 2015). Originally known as the “Barker hypothesis,” this theory described the association between low birth weight and the development of cardiovascular disease in adulthood (Barker, 1990). The hypothesis proposes that undernutrition early in life increases susceptibility to adult-onset metabolic syndrome, including obesity, high blood pressure, dyslipidemia, and insulin resistance (Barker, 1990; Barker et al., 1990; Lecoutre et al., 2021). The theory was later renamed the Developmental Origins of Health and Disease (DOHaD) Hypothesis to include the influence of overnutrition (obesity) and to extend the critical exposure window from gamete formation to postnatal development (Wadhwa et al., 2009).

The DOHaD hypothesis explains the relationship between low or high birth weight and the increased risk for developing metabolic syndrome in adulthood. Defined as a full-term birth weight less than <2.5 kg, low birth weight can be due to intrauterine growth restriction (IUGR), placental insufficiency, or maternal undernutrition. Approximately 15% of births worldwide, or more than 20 million infants each year, fall into this category (Cutland et al., 2017). Changes in metabolism in the low-birthweight fetus are adaptive to survive in the nutrient-poor *in utero* environment (Fall, 2013; Bhowmik et al., 2019). Ironically, when exposed to an obesogenic environment later in life, these same adaptations increase the risk of the offspring to metabolic disorders (Yajnik and Yajnik, 2020).

Parental obesity likewise predisposes the offspring to develop metabolic syndromes in adulthood. For instance, several human observational studies suggest maternal obesity is associated with metabolic disease in the offspring (Godfrey et al., 2017). Additional cohort studies indicate that parental Body Mass Index (BMI) predicts the BMI in the offspring (O’Callaghan et al., 1997; Rooney et al., 2011; Laitinen et al., 2012; Kaseva et al., 2018). The underlying mechanisms have been experimentally explored in animal models, that include rodents, sheep, and non-human primates (Reynolds et al., 2017). Studies in rodents and sheep demonstrate that feeding a high-fat diet during the periconceptional period and throughout gestation predisposes the offspring to develop metabolic syndrome (Rattanatray et al., 2010; Tessier et al., 2013; Lecoutre et al., 2016; Akhaphong et al., 2021). Lactation is another critical period when developmental programming is sensitive to overnutrition (Srinivasan and Patel, 2008). For example, a recent meta-analysis of multiple human observational studies reveals that breastfeeding is inversely associated with childhood obesity (Qiao et al., 2020). In the rat model, reducing litter size results in over-nourishment of pups during lactation and predisposes the offspring to hyperphagia, obesity, and metabolic syndrome later in life (Patel and Srinivasan, 2011)).

Understanding how parental obesity alters metabolic programs, predisposing the offspring to adult-onset metabolic syndrome, is key to breaking this cycle (Fall, 2013; Wells et al., 2020). In this review, we explore, from a developmental perspective, how parental obesity influences metabolism in the offspring. We draw from studies of human populations and experimental animal model systems revealing critical development processes impacted by obesity that contribute to long-term alterations in metabolism. These encompass gamete formation, placentation, adipogenesis, pancreas development, and brain appetite control circuits.

## Adiposopathy: Anatomic, endocrine, and metabolic dysfunction of adipose tissue in obesity

### Obesity, adiposity, and adiposopathy

While obesity is linked to metabolic disease, not all individuals with obesity develop metabolic syndrome (Neri and Edlow, 2016; Pétursdóttir Maack et al., 2020). Shortcomings in the metrics used to define obesity contribute to this discrepancy (Borga et al., 2018). For instance, obesity is often determined based on BMI, calculated using a patient’s height and weight, but does not differentiate between muscle, adipose tissue, or the distribution of adipose tissue (Liu et al., 2010; Rospleszcz et al., 2019). Adiposity is suggested as a measure of obesity as it excludes muscle mass and incorporates total and compartment-specific measurements of the adipose tissue (Borga et al., 2018). However, neither BMI nor adiposity can fully account for adipose tissue dysfunction. Adiposopathy, or “sick fat,” is a term that describes adipose dysfunction that comprises multiple histopathologic and molecular changes (Bays, 2011). The physiologic and endocrine changes that occur with adiposopathy include changes in the distribution of adipose tissue, inflammation, lipotoxicity, fibrosis, insulin resistance, and changes in the secretion of adipokines and extracellular vesicles (EVs) from adipose tissue. These measures have become more relevant as predictors of obesity-related metabolic disease over the past several years than hypertrophy of adipose tissue (Mundi et al., 2010; Pincu et al., 2022).

**FIGURE 1:**
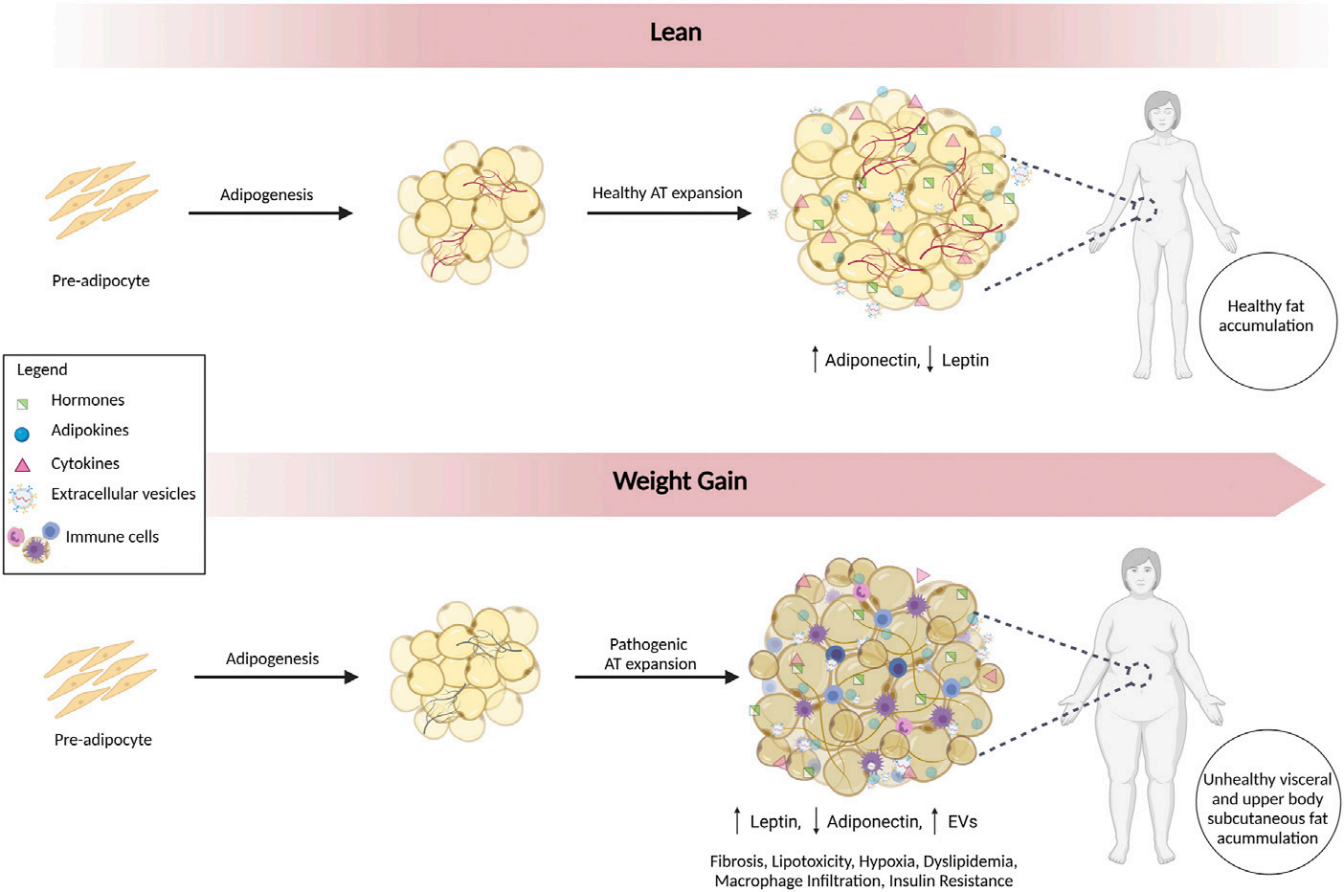
Alterations in Adipose Tissue in Adiposopathy. Adipogenesis involves the differentiation of adipocytes from pre-adipocytes. In lean individuals, healthy accumulation of subcutaneous fat predominates. With adiposopathy, WAT becomes dysfunctional, and expansion is accompanied by inflammation, fibrosis, lipotoxicity, hypoxia, dyslipidemia, macrophage infiltration, insulin resistance, dysregulated secretion of adipokines, and EVs. Fat accumulation occurs in metabolically unhealthy visceral and upper body subcutaneous depots.

Visceral fat accumulates around the intra-abdominal organs, and subcutaneous fat accumulates under the skin. Visceral adiposopathy contributes to metabolic syndrome, whereas subcutaneous accumulation is generally less harmful (Hoffstedt et al., 2010; Chait and den Hartigh, 2020). Furthermore, lower body subcutaneous fat is healthier with greater insulin sensitivity, and upper body subcutaneous fat is more insulin resistant (Chait and den Hartigh, 2020). The two main types of adipose tissue most relevant to obesity are brown adipose tissue (BAT) and white adipose tissue (WAT). BAT generates heat by burning calories, whereas WAT storages excess energy (Chait and den Hartigh, 2020). The mass and metabolic activity of BAT decrease with age and weight (Alcalá et al., 2019; Parra-Peralbo et al., 2021). With obesity, lipid accumulation and mitochondrial dysfunction shift the function of BAT from thermogenesis to lipid storage (Shimizu and Walsh, 2015). WAT also becomes dysfunctional **(** Figure 1). The energy storage capacity of WAT can expand by hyperplasia (cell number increase) or by hypertrophy (increased cell size). Hyperplasia involves the differentiation of adipocytes from pre-adipocytes in a process called adipogenesis. In contrast, hypertrophy of WAT results in increased release of inflammatory cytokines (e.g., TNF-α and IL-6) that promote insulin resistance (Makki et al., 2013). Hypertrophy of WAT with adiposopathy is accompanied by fibrosis, macrophage infiltration, hypoxia, and dysregulated secretion of adipokines and EVs, creating a pro-inflammatory environment (Ibrahim, 2010; Laforest et al., 2015).

Although excessive adipocyte hypertrophy is implicated in obesity/adiposopathy, recent studies suggested that adipocyte size and cell number can vary between individuals with obesity (Mundi et al., 2010; Suárez-Cuenca et al., 2021). The hypothesis that adipocyte size is a good predictor of the metabolic complications of obesity has been tested in animal models and human studies. In a lipodystrophic mouse model, reduced adipocyte number and size were associated with glucose intolerance, insulin resistance, increased serum triglycerides, and pro-inflammatory mediators (Sivasami et al., 2019). In patients with controlled obesity, small adipocytes are associated with inflammation in subcutaneous adipose tissue. This association was stronger in insulin-resistant than insulin-sensitive individuals (McLaughlin et al., 2007; McLaughlin et al., 2010). Moreover, Suaréz-Cuenca et al. (2021) described that adipocyte morphology and source are differentially related to insulin, adiponectin, blood glucose, and HbA1C levels in patients with obesity.

These data suggest that additional factors, not just adipocyte hypertrophy vs. hyperplasia, must be considered to characterize someone’s risk of obesity-related metabolic disease. One must characterize not just the size and distribution of adipose tissue but also adipose tissue function to understand the metabolic risks of obesity. Some have described the histopathologic and molecular changes in adipose tissue that correspond to the severity of obesity-related disease in individuals can be called “Obesity-related Adipose tissue Disease” or “OrAD.” These changes include adipocyte hypertrophy, inflammatory changes in the tissue, increasing fibrosis, and changes in the distribution of adipose tissue (Pincu et al., 2022). Accumulating dysfunctional ectopic visceral fat is metabolically harmful and is associated with insulin resistance, dyslipidemia, and type 2 diabetes (Neeland et al., 2019).

### Changes in endocrine function of adipose tissue in obesity

Adipose tissue not only stores energy but is an active endocrine organ that secretes autocrine and paracrine factors to influence energy metabolism throughout the body (Funcke and Scherer, 2019). Adiposopathy is associated with increased leptin and decreased adiponectin, contributing to metabolic disease (Frühbeck et al., 2019; Longo et al., 2019). In lean individuals, leptin secretion from WAT increases after feeding to signal satiety and control energy balance by regulating glucose and fat metabolism. However, with adiposopathy, prolonged elevated leptin levels and reduced circulating soluble leptin receptors results in the development of a leptin-resistant state (Ogier et al., 2002; Holm et al., 2011). In addition to regulating satiety, leptin acts as an inflammatory cytokine to modulate the immune response, and the increased leptin levels with adiposopathy foster low-grade chronic inflammation (La Cava, 2017; Kumar et al., 2020).

Conversely, adiponectin is a protective adipokine and has an inverse relationship with insulin resistance, visceral adiposity, and inflammatory markers (Booth et al., 2016). Adiponectin regulates glucose and fat metabolism but is anti-inflammatory (Fu et al., 2005). Low adiponectin levels are driven by increased visceral adiposity and are associated with chronic low-grade inflammation, with elevated TNFα, C-reactive protein, and IL-6 (Longo et al., 2019).

### Changes in extracellular vesicles in adiposopathy

EVs are another important secreted factor in adiposopathy (Camino et al., 2020; Huang and Xu, 2021). EVs are a heterogeneous group of cell-derived membranous particles with essential roles in intercellular communication. EVs contain proteins, lipids, and microRNAs that regulate the metabolism of multiple organs, including the liver, skeletal muscle, pancreas, and brain (Ferrante et al., 2015; Whitham et al., 2018; Barberio et al., 2019; Pérez-González et al., 2020). The contents and release of EVs are dramatically altered with adiposopathy (Akbar et al., 2019). Differences in miRNA contents of EVs are found in patients with obesity that can be improved with gastric bypass surgery (Koeck et al., 2014; Ferrante et al., 2015; Hubal et al., 2017). Interestingly, insulin resistance was one of the major pathways targeted by miRNAs in these EVs. Adiposopathy changes in EVs are likely functional; for instance, adipose-derived EVs from obese mice can stimulate inflammation and insulin resistance in lean mice (Deng et al., 2009). Conversely, EVs from lean mice improve insulin sensitivity in obese mice (Ying et al., 2017). Moreover, plasma EVs from women with obesity induce impaired insulin-stimulated glucose uptake in a human adipocyte cell line (Mleczko et al., 2018). These observations support the hypothesis that changes in the quantity and content of EVs are likely important mediators of dysfunction and inflammation that occur with adiposopathy (Ferrante et al., 2015; Huang-Doran et al., 2017).

Together, the changes in levels of EVs and adipokines that occur with adiposopathy play a central role in developing insulin resistance, type 2 diabetes, glucose intolerance, cardiovascular disease, and dyslipidemia (Landecho et al., 2019). As described in the next section, during pregnancy, these adiposopathy-associated metabolic changes collide with metabolic adaptations that prepare the mother for gestation and lactation. Thus, pregnancy-associated metabolic adaptations become pathological in women with obesity, altering not only the mother’s metabolism but the long-term metabolism of the developing fetus.

## Metabolic and endocrine changes in lean pregnancies

**FIGURE 2:**
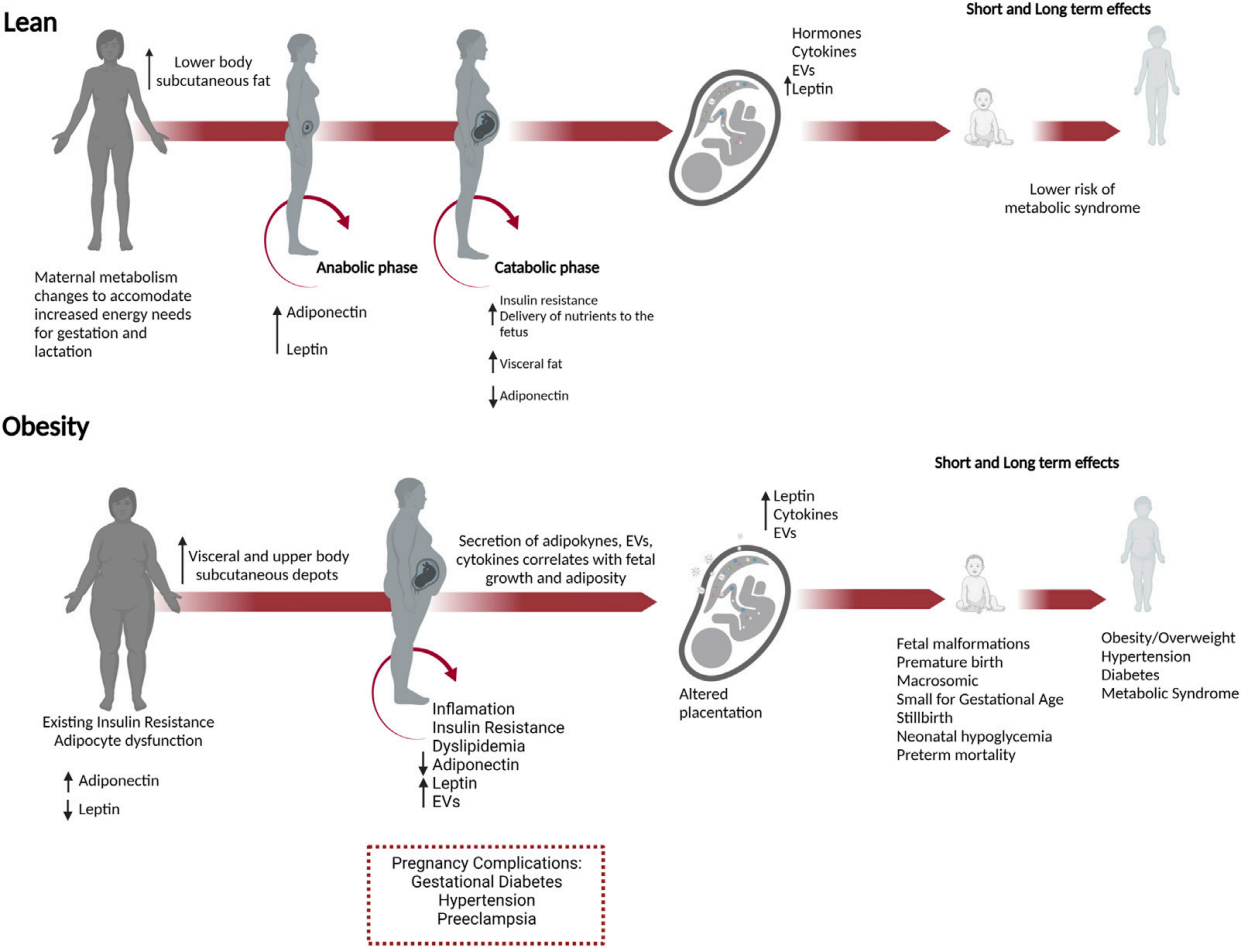
Obesity in pregnancy predisposes to short and long-term adverse outcomes in both the mother and offspring. Necessary metabolic adaptations occur during pregnancy that can go awry in women who enter pregnancy as overweight or obese. In lean pregnancies, maternal metabolism changes in anticipation of increased energy needs for gestation, and lactation. In the anabolic phase, increased insulin sensitivity and adiponectin promote the expansion of fat stores in lower body subcutaneous depots. In the catabolic phase during the second half of pregnancy, decreased adiponectin and insulin resistance occur to meet the increased energy needs and facilitate increased nutrient delivery to the developing fetus. These changes are managed in lean pregnancies and do not progress to metabolic dysfunction. In women with obesity, preexisting adipocyte dysfunction and insulin resistance result in fewer reserves for fat expansion and β cell adaptions. Visceral and upper body subcutaneous depots predominate, and fat is metabolically unhealthy with increased release of leptin, EVs, and decreased adiponectin. In lean women, lower body subcutaneous fat preferentially expands, whereas, in pregnancies with obesity, the growth of metabolically unfavorable visceral and upper body subcutaneous depots predominates. These changes have a greater likelihood of causing metabolic dysfunction in the mother, inducing insulin resistance, gestational diabetes, and hypertension. In the conceptus, placental dysfunction develops with increased leptin, cytokines, and EVs secretion, nutrient transporter expression. Together these changes increase fetal growth and adiposity, predisposing the offspring to metabolic syndrome (obesity, hypertension, and diabetes) in adulthood.

To understand how metabolism is altered in pregnancies with obesity, one must first appreciate the normal metabolic changes that occur during pregnancy (Figure 2). In lean pregnancies, maternal metabolism changes to accommodate increased energy needs for gestation and lactation. Lower body subcutaneous fat preferentially expands, but visceral fat later increases (Trivett et al., 2021). These changes occur in two phases (Zeng et al., 2017; Parrettini et al., 2020). In the first half of pregnancy, the anabolic phase enhances insulin sensitivity and expands fat stores in preparation for increased energy needs and lactation. The catabolic phase is characterized by insulin resistance which facilitates enhanced delivery of glucose and lipid to the developing fetus (Parrettini et al., 2020). This shift in metabolism results from changes to pancreatic β cells, liver hepatocytes, and adipocytes and in response to hormones, cytokines, and EVs secreted from the placenta and adipose tissue. (Jayabalan et al., 2017; Kampmann et al., 2019). Moreover, β cell mass, insulin secretion, and hepatic gluconeogenesis increase during pregnancy to enhance maternal weight gain and provide energy to the developing fetus (Banerjee, 2018; Salazar-Petres and Sferruzzi-Perri, 2022).

During human pregnancy, weight gain and increased fat mass results in elevated leptin secretion from both maternal adipocytes and the placenta (Masuzaki et al., 1997; Pérez-Pérez et al., 2018). These changes lead to increased leptin levels in the first and second trimesters of pregnancy and then a return to pre-pregnancy levels before birth (Briffa et al., 2015). Leptin resistance is an adaptation that occurs during pregnancy that allows for increases appetite and weight gain (Augustine et al., 2008). Furthermore, leptin is essential for implantation and placentation and plays a vital role in the development of the fetus (Pérez-Pérez et al., 2018). Adiponectin is secreted by adipose tissue during pregnancy and promotes insulin sensitivity, stimulating glucose uptake in maternal skeletal muscle (Aye et al., 2013). Adiponectin levels increase in the anabolic phase of pregnancy, supporting maternal weight gain, and then decrease during the catabolic phase, facilitating the allocation of nutrients to the fetus (Catalano et al., 2006; Mazaki-Tovi et al., 2007).

EVs are involved in maternal-fetal crosstalk during pregnancy, mediating an array of processes necessary for a successful pregnancy, including implantation, vascularization, decidualization, placentation, parturition, and modulation of the maternal immune response and metabolism (Jayabalan et al., 2019b; Bai et al., 2021; Condrat et al., 2021). In lean pregnancies, EVs are secreted from the placenta to influence maternal physiology and also signal from mother to fetus by interacting with the placenta and possibly the developing fetus (Czernek and Düchler, 2020). The total number of circulating EVs increases during pregnancy by approximately 50-fold (Salomon et al., 2014; Nakahara et al., 2020). Human placental-derived EVs are detected in maternal plasma as early as 6 weeks of gestation, with levels continuing to increase during gestation (Sarker et al., 2014). EVs may also reach the developing fetus. For instance, Cre-containing exosomes injected into the maternal circulation of cre-reporter mice, resulting in the recombination of the reporter in fetal membranes and the placenta (Sheller-Miller et al., 2019). Another study shows that DiI-labeled adipocyte-derived exosomes injected into the tail vein of pregnant mice can be found in the placenta and heart of embryos (Liu et al., 2021).

## Maladaptive changes in metabolism in pregnancies with obesity

Numerous metabolic and physiologic adaptations occur during pregnancy to ensure the delivery of sufficient nutrients to the developing fetus and prepare the mother for delivery and breastfeeding. These changes can become pathogenic in women who enter pregnancy as overweight or with obesity (Figure 2). They adversely affect insulin resistance, glucose, and lipid homeostasis in the mother and contribute to adverse pregnancy outcomes, including gestational diabetes, hypertension, cesarean delivery, postpartum hemorrhage, stillbirth, and preeclampsia (Fitzsimons et al., 2009; Nelson et al., 2010; Felisbino-Mendes et al., 2014). Moreover, maternal obesity increases the risk for developing a range of health problems later in life for the offspring, including a tendency to become overweight or obese and to develop hypertension, type 2 diabetes, metabolic syndrome, asthma, and neurocognitive disorders (Neri and Edlow, 2016; Kelly et al., 2020; Chang et al., 2021).

In pregnancies with obesity, growth of metabolically unfavorable visceral and upper body subcutaneous depots predominates (Ehrenberg et al., 2003; Straughen et al., 2013). Lean women can better accommodate the expansion of existing adipocytes, but the expansion of adipocytes in women with obesity is limited. Adipocyte dysfunction exacerbates the low-grade inflammation, insulin resistance, and dyslipidemia that normally occur during pregnancy, affecting placentation and contributing to the development of gestational diabetes (Figure 2; Trivett et al., 2021; Salazar-Petres and Sferruzzi-Perri, 2022). Placental dysfunction in pregnancies with obesity further contributes to complications, influencing the metabolic state of the mother through the release of hormones, cytokines, and EVs (Jayabalan et al., 2017; Kampmann et al., 2019).

### Maladaptive changes in adipokines and EVs secretions in pregnancies with obesity

While changes in adipokines and EVs are responsible for altering maternal metabolism to support pregnancy, in pregnancies affected by obesity, these changes contribute to metabolic dysfunction aggravating insulin resistance, hyperlipidemia, and inflammation (Christian and Porter, 2014; Corrales et al., 2021). Elevated leptin in pregnancies with obesity alters placentation, promotes inflammation, increases the risk for gestational diabetes, and correlates with increased fetal adiposity (Masuzaki et al., 1997; Pérez-Pérez et al., 2018). Conversely, reduced adiponectin further contributes to insulin resistance and gestational diabetes in both humans and rodents (Aye et al., 2013; Haghiac et al., 2014). For instance, in an obese rodent model, lower adiponectin levels in pregnancy increase the uptake of glucose and other nutrients by the placenta, that can be reversed by supplementing pregnant obese mice with adiponectin improving placental function, birthweight, and metabolism in the offspring (Aye et al., 2015; Paulsen et al., 2019; Vaughan et al., 2020).

Elevated levels of circulating placenta-derived EVs are found in both gestational diabetes and pregnancies with obesity (Salomon et al., 2016; Elfeky et al., 2017). Gestational diabetes is associated with increased levels of placenta-derived EVs which in turn increase the release of cytokines from endothelial cells contributing to fetoplacental endothelial dysfunction (Rice et al., 2015; Salomon et al., 2016; Sáez et al., 2018a, 2018b). Moreover, in gestational diabetes, EVs in maternal circulation are elevated with pathological changes in the composition and bioactivity of their contents that negatively affect energy production, inflammation, and metabolism (Salomon et al., 2016; Palma et al., 2022). For instance, differential expression of proteins related to insulin sensitivity is found in EVs in women with gestational diabetes (Jayabalan et al., 2019a). The miRNAs enriched in EVs from women with gestational diabetes target insulin and glucose regulatory pathways (Gillet et al., 2019). Moreover, placenta-derived EVs from pregnancies with gestational diabetes contain higher levels of inflammatory cytokines (Rice et al., 2015).

In summary, metabolic adaptations during pregnancy can become maladaptive in women with obesity. Changes in adiposity, adipokines, and EV composition and release contribute to the development of gestational diabetes and other pregnancy complications associated with obesity. These conditions negatively affect the metabolic programming of the offspring, predisposing them to metabolic syndrome later in life.

## Changes in developmental programming in the offspring from parents with obesity

Pathogenic changes in adipokines and EVs, hyperlipidemia, insulin resistance, and elevated glucose in pregnant mothers with obesity alter the developmental programming of multiple organ systems in the offspring. These changes have lifelong effects on metabolism by modifying the epigenome and altering gene expression. Epigenetic mechanisms play critical roles in fetal programming, predisposing offspring to metabolic syndrome (Barouki et al., 2012; Neri and Edlow, 2016; Li, 2018). Deviations in developmental programming induced by parental obesity occur as early as gamete formation and continue through lactation. In the placenta, maternal obesity leads to changes in gene expression which not only alter placental hormone production but increase inflammation and nutrient transport to the developing fetus (Huynh et al., 2015; Gallo et al., 2017; Kelly et al., 2020). Augmented delivery of nutrients, lipids, and glucose to the developing fetus modify developmental plasticity and epigenetic programming of fetal adipocytes and pancreatic β cells. Postnatally, changes in the development of appetite control circuits that control satiety regulation led to hyperphagia (McMillen et al., 2005). Epigenetic changes affect long-term lipid metabolism and energy expenditure in the liver and skeletal muscle in the offspring (Friedman, 2018). Together, these deviations in developmental programming permanently alter the metabolism of the offspring, predisposing them to metabolic syndrome later in life.

### Parental obesity modifies the epigenome

Epigenetic modifications are persistent and heritable changes in the DNA. They include DNA methylation, post-translational modifications of histones, and noncoding RNA such as microRNAs that regulate gene expression at both transcriptional and post-transcriptional levels (Skvortsova et al., 2018). DNA methylation requires the transfer of a methyl group from donors synthesized in the one-carbon metabolism cycle. Multiple micronutrient deficiencies are associated with obesity and can impact one-carbon metabolism affecting the availability of methyl donors required for epigenetic modifications providing a link between maternal diet and epigenetic regulation. For instance, obesity is associated with folate and B12 deficiency and elevated homocysteine in human populations (Sanchez-Margalet et al., 2002; Karatela and Sainani, 2009). In mouse models, feeding a high-fat diet alters epigenetic programming through the reduced availability of methyl donors (Vucetic et al., 2010). Differentiation of human adipocytes in low vitamin B12 conditions alters the expression of miRNAs involved in adipocyte differentiation and function altering expression of adipocyte-derived circulating miRNAs that target transcripts involved in adipogenesis (Adaikalakoteswari et al., 2017).

Vitamin D is another critical micronutrient with epigenetic consequences. Vitamin D deficiency is common in individuals with obesity as higher adiposity reduces circulating vitamin D (Hyppönen and Boucher, 2018). Children of vitamin D deficient mothers and offspring of vitamin D deficient mice have higher BMIs (Nascimento et al., 2013; Morales et al., 2015; Daraki et al., 2018). Moreover, vitamin D deficiency in childhood is linked to increased adiposity and metabolic syndrome in adulthood (Hyppönen and Boucher, 2018).

The association of obesity with imprinting syndromes such as Prader-Willi syndrome (PWS) and Albright hereditary osteodystrophy further implicates epigenetics in obesity (Shapira et al., 2005; Butler, 2009). Moreover, essential genes that regulate fetal size are regulated by imprinting. These include components of the insulin-like growth factor signaling pathway such as *IGF2,* expressed from the paternal allele to promote growth, and *IGF2R,* expressed from the maternal allele to inhibit growth (Smith et al., 2006). Several genome-wide association studies suggest that genetics likely only accounts for modest proportions of obesity at the population level (Loos, 2012). In this regard, epigenetic, rather than genetic mechanisms, are likely important in influencing developmental programming.

### Parental obesity alters gametogenesis and preimplantation development of the offspring

From oocyte/sperm maturation to pre-and post-implantation development, the periconceptional period is a critical time in development where parental obesity can influence epigenetic changes with long-term metabolic consequences on the offspring (Huypens et al., 2016; Nicholas et al., 2016). Analysis of *in vitro* fertilization (IVF) data in humans and experiments in mice indicate that oocyte quality is influenced by maternal obesity, which impacts the metabolism of the offspring through epigenetic changes (Igosheva et al., 2010; Luzzo et al., 2012; Leary et al., 2015; Fleming et al., 2018). Transfer of IVF embryos from high-fat diet-fed mice with obesity to lean surrogates results in greater susceptibility to obesity and diabetes (Huypens et al., 2016). Interestingly, metabolic changes were more prominently inherited through maternal than paternal gametes, and female offspring were more susceptible than their male siblings (Huypens et al., 2016). The conclusion that epigenetic changes mediate these results is further supported by transgenerational inheritance, where the F2 generation (grandchildren) of the grandparents (F0) exposed to a high-fat rodent diet during gamete production were predisposed to metabolic syndromes (Anwer et al., 2022). Additional evidence of the importance of the preimplantation exposure window on the metabolism of the offspring comes from studies of IVF in humans. For instance, children conceived by IVF are at increased risk for imprinting disorders and metabolic diseases, including high blood pressure, insulin resistance, hyperlipidemia, and obesity (Ceelen et al., 2008; Scherrer et al., 2012; Valenzuela-Alcaraz et al., 2013; Gkourogianni et al., 2014). Similarly, studies in mice showed that the culture of preimplantation embryos before embryo transfer resulted in long-term metabolic changes in the offspring (Watkins et al., 2007).

Epigenetic changes in the sperm also contribute to the transgenerational inheritance of obesity and metabolic disorders (Craig et al., 2017). In humans, paternal obesity is associated with effects on placental development and gene expression in the liver and pancreas (Binder et al., 2012, 2015). Similarly, in rodent models, paternal obesity is linked to changes in DNA methylation and altered gene expression in the fetal liver and pancreas (Carone et al., 2010; Wei et al., 2014). Additional studies in rats connect paternal obesity to altered gene expression in sperm and long-term changes in the metabolism of female offspring (Ng et al., 2010, 2014). For instance, feeding male rats a high-fat diet alters DNA methylation patterns in the progeny, providing further evidence that paternal obesity is transmitted through epigenetic changes (Sinclair and Watkins, 2013; De Castro Barbosa et al., 2016; Fleming et al., 2018; Deshpande et al., 2021).

### Maternal obesity and changes in the placenta

**FIGURE 3:**
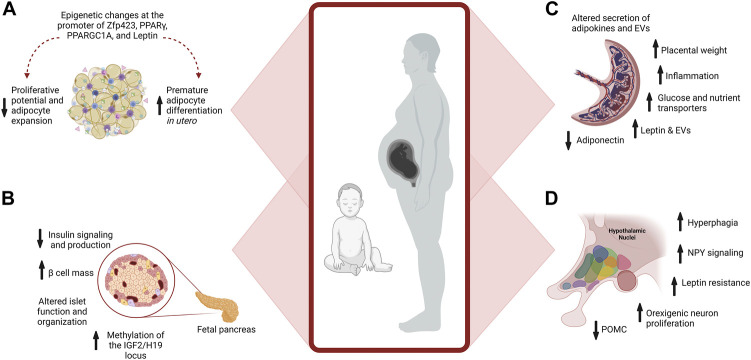
Maternal Obesity Alters Placenta, Adipocyte, and β cell Development. **(A)** Maternal obesity promotes premature differentiation of adipocytes, which depletes progenitors available for expansion in adulthood when excess energy storage may be needed. Epigenetic changes at the promoter of multiple genes further affect developmental plasticity. **(B)** Pancreatic development is influenced by maternal obesity. Exposure to hyperinsulinemia during fetal development increases Islet size and β cell mass, reducing insulin signaling and production. Epigenetic changes also occur, such as increased methylation of *IGF2* in the pancreas, influencing growth and function. **(C)** In the placenta, maternal obesity alters the placental secretion of adipokines and EVs, increases inflammation, and placental weight, and modifies nutrient transport to the developing fetus. **(D)** Overfeeding postnatally alters hypothalamic circuits and food intake.

The placenta is essential for exchanging nutrients and waste between maternal and fetal circulations. It secretes hormones that support pregnancy and acts as a sensor, altering the expression of nutrient transporters to align the uptake of nutrients with maternal metabolism (Glazier et al., 1997; Constância et al., 2002, 2005; Dilworth et al., 2010; Huynh et al., 2015; Myatt and Maloyan, 2016). Significantly, maternal obesity and gestational diabetes induce multiple pathogenic changes in the placenta. These include increased placental weight, inflammation, and altered adipokine and EV secretions (Figure 3) (Aye et al., 2014; Myatt and Maloyan, 2016; Salomon et al., 2016; Howell and Powell, 2017). Additionally, hyperglycemia in gestational diabetes induces inflammation of the fetoplacental vasculature (Rice et al., 2015; Salomon et al., 2016; Elfeky et al., 2017; Echeverria et al., 2020).

In a vicious cycle, placental dysfunction in obesity and gestational diabetes paradoxically leads to increased expression of placental glucose transporters, further increasing glucose uptake (Salomon et al., 2016; Plows et al., 2018). Increased insulin resistance affects early placental growth, altering gene expression, including genes that regulate lipid metabolism and mitochondrial activity (Parrettini et al., 2020). Gestational diabetes alters the expression of nutrient transporters in the placenta bringing excess nutrients to the developing fetus and promoting excessive fetal growth (Jansson et al., 2002, 2013; Castillo-Castrejon and Powell, 2017). Adipokines also regulate the expression of nutrient transporters in the placenta, and altered adipokine expression in obesity further contributes to placental dysfunction (Howell and Powell, 2017; Jayabalan et al., 2017; Corrales et al., 2021). Leptin increases nutrient availability to the fetus by stimulating the placental amino acid transport system promoting overnutrition of the fetus through increased transport of nutrients (Jansson et al., 2002, 2003; Von Versen-Höynck et al., 2009). Conversely, lower adiponectin levels in pregnancies with obesity upregulates glucose and amino acid transporters, resulting in increased transport of nutrient delivery by the placenta to further stimulate fetal growth (Jones et al., 2010; Rosario et al., 2012; Aye et al., 2013; Duval et al., 2016).

### Maternal obesity alters adipocyte development in the offspring

Maternal obesity alters the developmental plasticity, distribution, and composition of adipose tissue in the offspring, predisposing them to adiposopathy in adulthood (Lecoutre and Breton, 2015; Lecoutre et al., 2021). Adipocytes are derived from multipotent mesenchymal stem cells, which become committed to the adipocyte lineage as pre-adipocytes. This commitment is influenced by a series of coordinated events that involve transcription factors, cofactors, and numerous signaling pathways (Rosen and MacDougald, 2006). Pre-adipocytes undergo clonal expansion and differentiate into functional adipocytes under the influence of leptin and adiponectin (Shin et al., 2020). These events occur between the 14th to 24th weeks of gestation in humans and mid-gestation to birth in rodents (Poissonnet et al., 1984; Lecoutre et al., 2018). Adipocyte numbers are typically established during childhood and adolescence but can expand if needed to store excess energy (Spalding et al., 2008). However, this is probably not absolute, since pre-adipocytes from elderly humans were found to differentiate *in vitro* (Hauner et al., 1989).

Our understanding of adipocyte development comes from both *in vitro* and animal studies and data on depot-specific aspects of differentiation are limited (Rosen and MacDougald, 2006). As such, much is still unknown about the precise developmental origins of fetal fat depots. It is proposed that subcutaneous and visceral adipocytes are specified at different stages of development, with subcutaneous fat specified in the fetus and visceral fat in the neonate (Desoye and Herrera, 2021). Furthermore, it is suggested that subcutaneous and visceral depots arise from distinct lineages (Ying and Simmons, 2021).

One-way maternal obesity influences fetal adipocyte development is by promoting premature differentiation of pre-adipocytes, depleting progenitors available for expansion in adulthood (Lecoutre et al., 2021). Depletion of progenitors alters the potential of the adipocyte lineage to expand in adulthood when excess energy storage may be needed (Berry et al., 2016). Reduced proliferative potential of adipocyte progenitors results in pathogenic expansion of adipocytes (Lecoutre et al., 2021) (Figure 3). Late gestation and lactation are critical times for expanding adipocytes and are periods when developmental programming is highly sensitive to overnutrition (Srinivasan and Patel, 2008).

Epigenetic alterations influence adipocyte remodeling in the offspring of mothers with obesity, and the persistence of these marks is a significant contributor to the long-term consequences of overnutrition during pregnancy (Lecoutre et al., 2021). For instance, increased leptin levels in the offspring of rats fed a high-fat diet during pregnancy are associated with the persistent epigenetic remodeling at vital regulatory regions of the leptin gene in adipocytes (Lecoutre et al., 2016, 2017). In humans, increased methylation of specific epigenetic sites in cord blood is observed in offspring of mothers with obesity and is correlated with adiposity in the offspring (Sharp et al., 2015). In rodents, maternal obesity alters expression via epigenetic changes at the promoters of key transcription factors involved in adipogenesis, including Zfp423, PPARγ, and PPARGC1A (Gemma et al., 2009; Borengasser et al., 2013; Yang et al., 2013; Lecoutre et al., 2018). Altered expression of microRNAs is another epigenetic modification that impacts adipose tissue metabolism in offspring of pregnancies with obesity. For instance, overexpression of miR-126 in adipocytes of male mice offspring targets the downregulation of insulin receptor substrate-1 (IRS-1), modulating insulin signaling (Figure 3) (Fernandez-Twinn et al., 2014; De Almeida-Faria et al., 2021). These epigenetic changes promote premature differentiation, attenuating the expansion of adipocytes and favoring metabolically adverse modes of expansion in adulthood.

### Obesity alters pancreatic β cells development in the offspring

Changes in the developmental plasticity of insulin-producing pancreatic β cells are another critical long-term maladaptation in response to maternal obesity (Lecoutre et al., 2021). Insulin is produced from β cells of the pancreas in response to increased blood glucose levels and facilitates glucose uptake into cells. This feedback loop is essential for maintaining blood glucose levels in a healthy range.

Islets of the pancreas have a complex architecture and are composed of four cell types (α, β, D and F cells). α cells secrete glucagon, and β cells secrete insulin. β cells are highly susceptible to the deleterious effects of maternal obesity and gestational diabetes (Lecoutre et al., 2021). The development of β cells of the pancreas is best characterized in rodent models. The differentiation phase of β cell development occurs in the mouse embryo between embryonic days (E) E9.5 and E12.5 with the specification of multipotent endocrine progenitor-precursors from the embryonic endoderm (O’Dowd and Stocker, 2013). From E12.5 to birth, these progenitors differentiate into various endocrine cells, including β cells. The maturation phase occurs postnatally when β cells are immature, plastic, and actively proliferate until weaning when they mature and produce insulin (Lecoutre et al., 2021).

Differentiation and maturation phases of β cell development are most susceptible to the deleterious effects of maternal obesity and gestational diabetes (Lecoutre et al., 2021). Maternal obesity alters β cell size, function, and organization within the islets of the offspring (Figure 3). These changes are influenced by the sex, timing, and degree of glucose dysregulation. Pancreatic islet cells are arranged in a highly stereotypical fashion essential for their function. In rodent models of maternal obesity and gestational diabetes, changes in β cell development and organization are found in the offspring that alter insulin production and glucose regulation into adulthood (Lecoutre et al., 2021). Similarly, in humans, islet hypertrophy and hyperplasia of β cells are found in newborns from mothers with gestational diabetes (Holemans et al., 2003).

Offspring from mice fed an obesogenic diet with high sugar and fat levels throughout gestation and lactation exhibited elevated fasting glucose and insulin resistance (Samuelsson et al., 2008). In another study, exposure to a high-fat diet during gestation but not lactation resulted in hyperinsulinemia and increased β cell mass in male offspring (Gregorio et al., 2013). In contrast, maternal obesity without diabetes resulted in altered β cell function, increasing the risk of gestational diabetes and type 2 diabetes in the female but not male offspring (Han et al., 2005). Furthermore, in mice, a maternal high-fat diet alters islet structure in the male offspring, impairs the insulin-signaling pathway, increases FOXO1 protein expression, and decreases IRS1, PI3K, p-Akt, Pd-1, and GLUT2 protein expression in the pancreas (Bringhenti et al., 2016). The imprinted *IGF2* locus is a critical developmental regulator of pancreas size and function and becomes hypermethylated in the pancreas of offspring from pregnant mice with gestational diabetes (Ding et al., 2012; Hammerle et al., 2020). These examples not only demonstrate the alterations in β cell development of the offspring induced by maternal obesity and the impact on glucose control in the offspring but also highlight the need for consideration of the diet used to induce obesity, the degree of glucose dysregulation, and sex differences of the offspring in rodent studies.

### Obesity and altered development of appetite control

**FIGURE 4:**
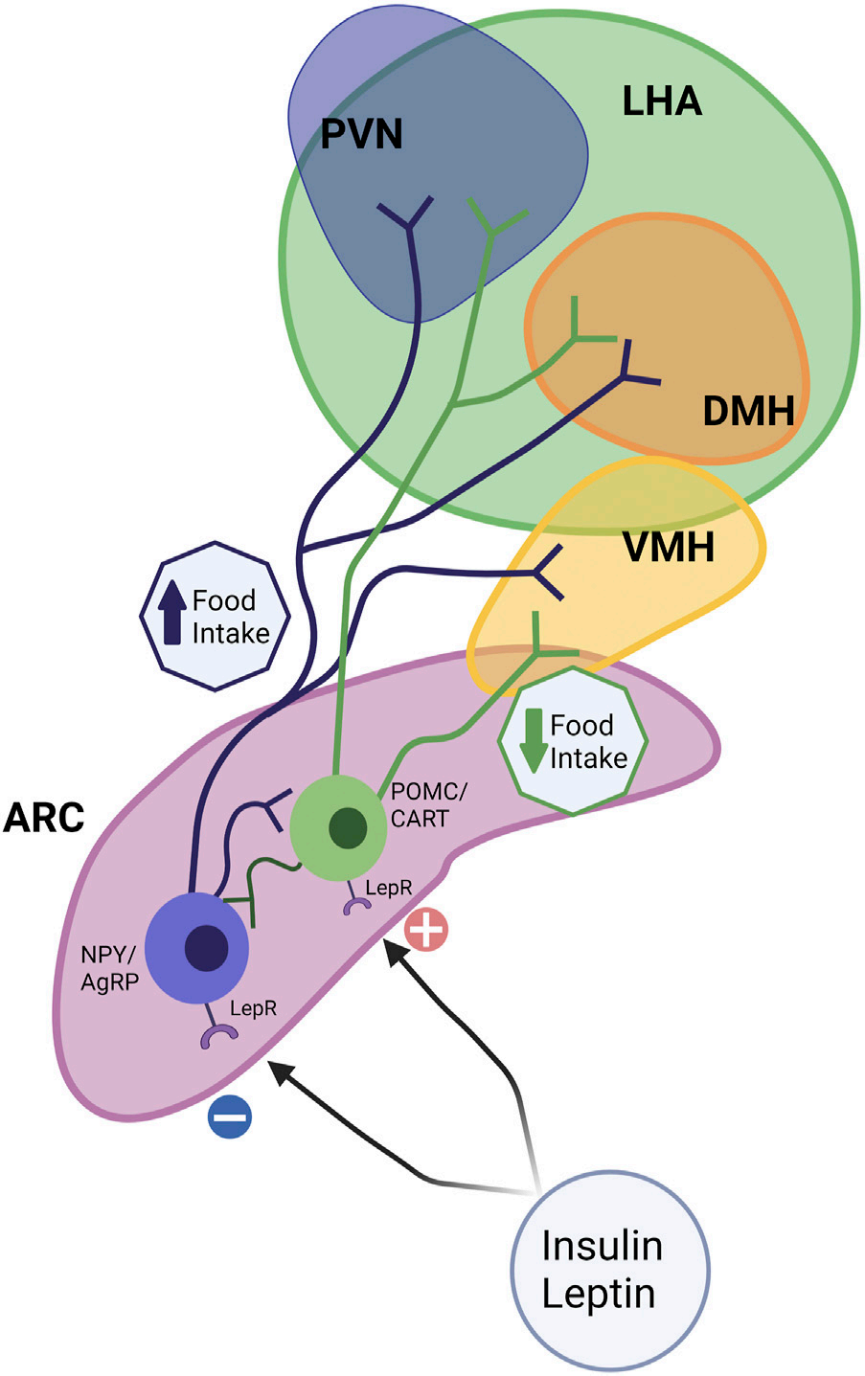
Hypothalamic circuits and Food Intake. The hypothalamus is considered the main integrator and processor of peripheral metabolic information. Among several hypothalamic nuclei such as the arcuate nucleus (ARC), paraventricular nucleus (PVN), the dorsomedial hypothalamus (DMH), ventromedial hypothalamus (VMH), and the lateral hypothalamic area (LHA), ARC and VMH are the predominant sites for the integration of signals. Hypothalamic nuclei control feeding and energy expenditure by the release of orexigenic (appetite-stimulating; neuropeptide Y [NPY] and agouti-related protein [AgRP]) and anorexigenic (appetite inhibiting; pro-opiomelanocortin [POMC] and cocaine- and amphetamine-regulated transcript [CART]) peptides. The ARC contains anorexigenic and orexigenic neurons, POMC/CART, and NPY/AgRP, which project extensively to other hypothalamic nuclei. Leptin and other signals upregulate anorexigenic neurons (POMC/CART) and reduce food intake. NPY neurons (orexigenic) are sensitive to peripheral nutrient and hormone fluctuations, such as insulin, and leptin, which promote food intake.

Maternal obesity and overfeeding postnatally have long-term consequences on appetite control in the offspring. Appetite is regulated centrally through a complex circuit involving the Hypothalamic-Pituitary-Adrenal Axis (HPA axis; Figure 4). The hypothalamus is the main integrator and processor of peripheral metabolic information. Hypothalamic nuclei control feeding and energy expenditure by the release of orexigenic (appetite-stimulating; neuropeptide Y [NPY], agouti-related protein [AgRP]) and anorexigenic (appetite inhibiting; pro-opiomelanocortin [POMC] and cocaine- and amphetamine-regulated transcript [CART]) peptides (Ross and Desai, 2014). There are several hypothalamic nuclei described in Figure 4, relevant to appetite control.

Together these nuclei form an integrated circuit (Figure 4) that regulates food intake and appetite, integrating with peripheral signals such as leptin and insulin to control appetite (for review, see McMillen et al., 2005). ARC can be considered a predominant site for the integration of signals. The ARC contains both anorexigenic (POMC/CART) and orexigenic (NPY/AgRP) neurons that project extensively to other critical hypothalamic nuclei, such as the PVN, DMH, and LHA (McMillen et al., 2005). αMSH, a POMC-derived peptide, acts on the melanocortin receptor to suppress food intake. In addition, leptin upregulates POMC expression and therefore limits energy intake. The neuropeptide CART, co-expressed by POMC neurons, also inhibits food intake (Schwartz, 2001). NPY neurons are sensitive to peripheral nutrient and hormone fluctuations, such as glucose, insulin, and leptin (Gali Ramamoorthy et al., 2015). The ARC also expresses AgRP, an endogenous antagonist of the anorexigenic receptors in the PVN and other hypothalamic regions (Woods et al., 1998) (Figure 4).

The development of orexigenic and anorexigenic hypothalamic neurons starts during fetal life and continues its growth and remodeling in the neonatal period (Kagotani et al., 1989). In rodents, POMC becomes detectable at gestational day E12.5, and NPY neurons first appear in the ARC at E14.5 (Kagotani et al., 1989). Neuronal postnatal development is different between rodents, humans, and nonhuman primates, as hypothalamic neural projections develop almost entirely during fetal life in primates (Koutcherov et al., 2002). After hypothalamic neuroprogenitor cells (NPCs) differentiate into neurons, those cells differentiate to specific neuronal phenotypes: orexigenic (NPY) or anorexigenic (POMC), for example. NPCs proliferation and differentiation processes are regulated by a series of neuroregulatory basic helix-loop-helix (bHLH) transcription factors, including Hes1, Neurogenin (Ngn3), and Mash1 (Kageyama et al., 2008; Desai et al., 2020). Insulin and leptin regulate adult appetite behavior, but they also have important roles in appetite neurogenesis. For example, neurons treated with insulin exhibit cellular growth, and axonal growth was stimulated by insulin in a rat fetal brain cell culture. Whereas leptin-deficient or -insensitive animals displayed decreased brain size and development (Ross and Desai, 2014).

The timing of terminal differentiation defines a critical period during lactation for appetite regulation. In this context, male offspring from mice fed an HFD during pregnancy and lactation showed reduced hypothalamic bHLH factors, pAMPK/AMPK, and POMC while increased AgRP with NPCs showing preferential differentiation towards NPY (Desai et al., 2020). Feeding rats an obesogenic diet before mating until weaning induces long-term hyperphagia in the offspring (Samuelsson et al., 2008). Further studies define the perinatal period as critical, where high-fat diet exposure increases embryonic hypothalamic orexigenic cell proliferation in the PVN and LHA (Chang et al., 2008). Moreover, a high-fat diet during the perinatal period was also associated with reduced hypothalamic POMC neurons in postnatal age p1 and 12 months old male mice (Desai et al., 2020). In rodents, maternal obesity increases post-weaning high-fat diet-induced hyperphagia, hypothalamic NPY signaling, and leptin resistance in the offspring through adulthood (Chen et al., 2009). Rodent studies demonstrate that reducing litter size during lactation stimulates overfeeding and alters hypothalamic circuits to promote hyperphagia, predisposing offspring to increased body weight later in life (Patel and Srinivasan, 2011). Additionally, feeding rat pups milk from dams with gestational diabetes increased NPY and decreased POMC neurons in the ARC contributing to hyperphagia and later-onset metabolic syndrome (Fahrenkrog et al., 2004). Together these studies highlight the influence of overfeeding during late gestation/lactation on developing hypothalamic circuits and long-term appetite regulation.

## Summary and future directions

**FIGURE 5:**
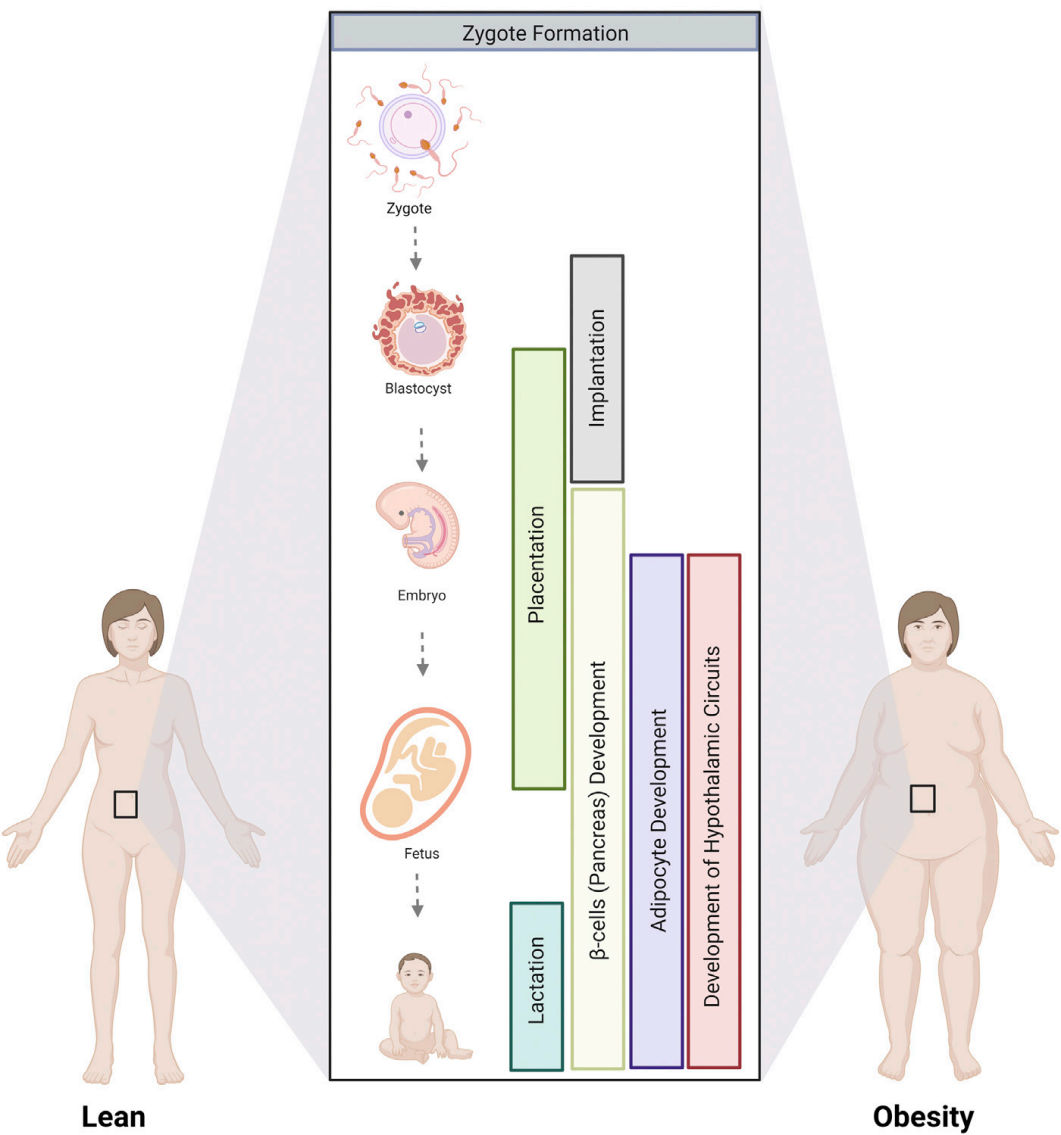
Critical stages where parental obesity influences developmental programming. The development of multiple tissues and organs involved in metabolism is altered by parental obesity from gamete formation until lactation. Critical stages involve gamete formation, placentation, and the development of adipocytes, the pancreas, and the hypothalamic circuits that control appetite that occurs during fetal and postnatal development.

With the growing obesity epidemic, the number of pregnancies affected is increasing, and so is the risk of metabolic disease in the offspring. Understanding how parental obesity alters metabolic programs in the progeny, predisposing them to adult-onset metabolic syndrome, is key to designing targeted interventions to break this cycle. This review synthesizes a broad literature on this topic to explore how parental obesity alters the development of the gametes, placenta, adipocytes, pancreatic β cells, and hypothalamic circuits that control feeding. We identify critical stages where parental obesity alters developmental programming (Figure 5). While not examined in isolation in all studies, select experiments in animal models demonstrate that exposure to parental obesity at distinct developmental phases is sufficient to alter certain aspects of the metabolism of the offspring. In humans, multiple stages of development are typically impacted, adding insult to injury to compound long-term changes in offspring metabolism.

Insulin resistance and glucose control are critical targets for intervention. In rodents, altering the carbohydrate and lipid composition of the maternal diet or increasing physical activity during pregnancy improves insulin resistance and glucose control in both mothers and their offspring (Vega et al., 2015; Gimpfl et al., 2017; Ramalingam et al., 2018; Rodrigo et al., 2022). Changes are most efficacious when started before pregnancy and continued through gestation (Stanford et al., 2015; Stanford et al., 2017; Harris et al., 2018; Summerfield et al., 2018; Kusuyama et al., 2020; Smits et al., 2021; Rodrigo et al., 2022). Humans are not always compliant with these changes, making modifying diet and exercise during pregnancy less successful (Dodd et al., 2014; Gimpfl et al., 2017; Rodrigo et al., 2022). One mechanism by which exercise could improve outcomes is by increasing circulating adiponectin, which affects the metabolism of the mother and the offspring by influencing placental vascularization, nutrient transport and fetal growth (Jones et al., 2010; Kelly et al., 2020; Mangwiro et al., 2018a; 2018b; Rosario et al., 2012; Son et al., 2019).

Adiponectin levels are reduced in pregnancies with obesity, and adiponectin supplementation may be an effective strategy to mitigate intergenerational effects. Supplementing pregnant dams with adiponectin counters the adverse effects of maternal obesity on placental function and fetal growth, ameliorating long-term metabolic syndrome in the offspring (Aye et al., 2015). Furthermore, adiponectin supplementation during gestation improves insulin sensitivity and reduces weight gain in the offspring of prenatal androgenized female adult offspring (Wu et al., 2021). Another study showed that administering adiponectin receptor agonist (AdipoRon) to pregnant diabetic rats improved insulin control in the mothers and long-term glucose tolerance in the offspring (Gázquez et al., 2021).

Another theme that emerges is the role of epigenetic changes in multiple organs that are essential for transmitting the long-term influence of parental obesity on the metabolism of the offspring. Obesity is associated with various micronutrient deficiencies, which are an attractive actionable target to alleviate some of these impacts of obesity. For instance, micronutrients involved in the one-carbon cycle generate the methyl groups that mediate epigenetic changes may be an actionable target. Supplementation with folic acid, Vitamin B12, and Vitamin D are promising strategies for reverting epigenetic modifications. Finally, postnatally, significant developmental programming of adipocytes, β cells, and hypothalamic circuits occurs. Targeted strategies delivered postnatally may prevent these changes from happening or even reverse some of the damage done *in utero*.
